# Breast hemangioma with difficulty in preoperative diagnosis: a case report

**DOI:** 10.1186/1477-7819-12-313

**Published:** 2014-10-14

**Authors:** Naotake Funamizu, Isao Tabei, Chikako Sekine, Azusa Fuke, Mitsuo Yabe, Hiroshi Takeyama, Tomoyoshi Okamoto

**Affiliations:** Department of Surgery, The Jikei University School of Medicine, Daisan Hospital, 4-22-1, Izumi honnmachi, Komae-city, Tokyo, 201-8601 Japan; Department of Surgery, The Jikei University School of Medicine, 3-25-8, Nishi-shimbashi, Minato-ku, Tokyo, 105-8461 Japan

**Keywords:** Breast hemangioma, Breast hemangiosarcoma

## Abstract

We report a rare case of breast hemangioma found in a 70-year-old Japanese female. Before seeking medical attention, the patient noticed a hard mass in her right breast but denied associated symptoms. A mammography revealed a well-circumscribed, highly dense, lobular nodule located in the middle inter portion of the right breast. To verify this finding, we used ultrasonography which revealed an irregular, iso-echoic nodule measuring 10 mm in the same portion. Based on these findings, we suspected a malignancy and performed a core needle biopsy. Unexpectedly, a histological examination of the biopsy displayed normal vasculature, adipose, and mammary tissues. In order to make an accurate diagnosis, the mass was surgically excised under general anesthesia and sent to pathology. Pathological findings of the mass were positive for breast hemangioma, and the patient has had no recurrence of the disease for the past 24 months.

**Electronic supplementary material:**

The online version of this article (doi:10.1186/1477-7819-12-313) contains supplementary material, which is available to authorized users.

## Background

Breast hemangioma is a rare, benign vascular tumor that accounts for only 0.4% of all breast tumors [1]. Preoperatively, hemangiomas of the breast are difficult to diagnose using conventional imaging modalities since they lack pathognomonic characteristics. We report the case of a 70-year-old Japanese female with a breast hemangioma.

## Case presentation

**Figure 1:**
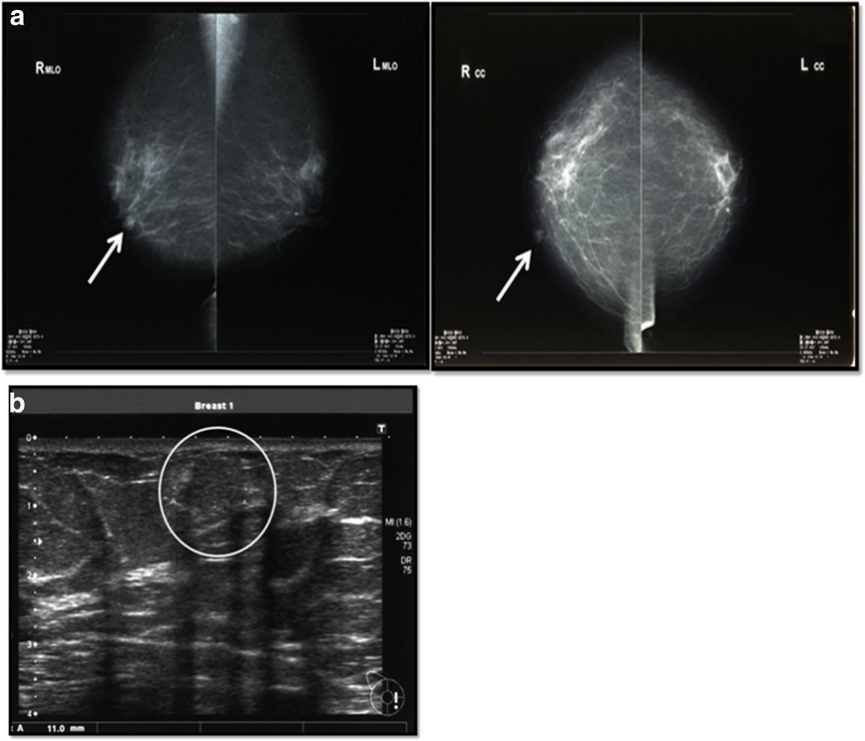
**A mammography revealed a circumscribed, high-density lobular nodule. a**. The nodule measured 1 cm in diameter and was located in the middle inter portion of her right breast. **b**. An ultrasonography (US) revealed a heterogeneous, iso-echoic mass with irregular margins measuring 10 × 12 mm in diameter. No accompanying posterior acoustical shadowing was observed.

A 70-year-old Japanese female consulted a local hospital upon noticing a hard mass in her right breast, and was later referred to our hospital for diagnosis and treatment. The patient denied a family history of breast cancer, while her physical examination revealed a firm, mobile mass measuring 10 mm. No axillary lymphadenopathy or nipple discharge was observed. A mammography (MMG) was performed in the standard craniocaudal and mediolateral oblique positions. The MMG demonstrated a well-circumscribed, highly dense, lobular mass in the middle inner portion of the right breast (Figure 1a). No associated calcifications were present.

**Figure 2:**
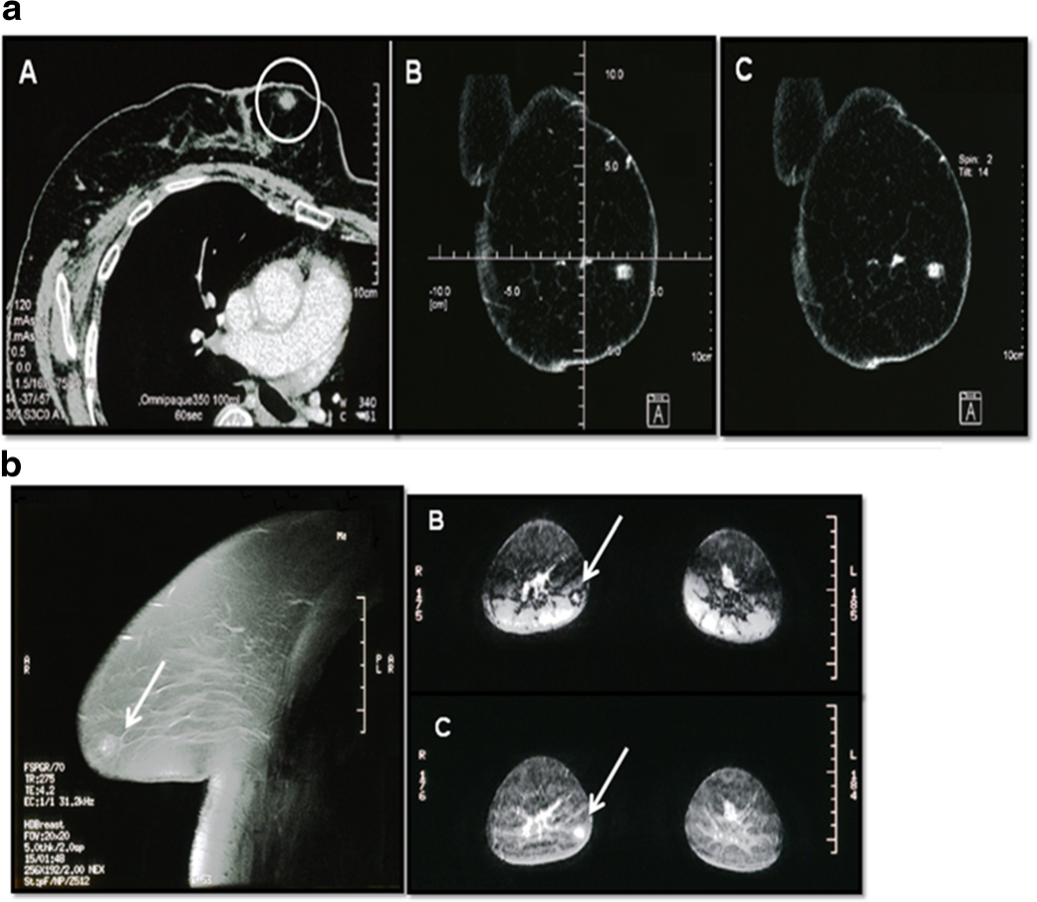
**Enhanced computed tomography and magnetic resonance imaging findings. a**. Enhanced computed tomography showed a well-enhanced nodule in both the early and late phase. **b**. Dynamic T-1 weighted image revealed a highly intense nodule in the right breast **(A)**. A subtraction image showed a hyper-intense nodule in early and late phases **(B, C)**.

We followed up the MMG with an US which revealed a poorly demarcated, iso-echoic lesion of 10 mm without any accompanying posterior echo attenuation (Figure 1b). Contrast-enhanced computed tomography (CT) showed a well-defined, enhanced mass at both the early and late phases (Figure 2a).

Following variations in results, the patient underwent dynamic contrast-enhanced magnetic resonance imaging (MRI). T1-weighted images of the tumor displayed high signal intensity, while the dynamic contrast-enhanced MRI demonstrated a heterogeneous enhancement in both the early and delayed phases (Figure 2b). Based on our MRI findings, a preoperative diagnosis of occult breast cancer was suspected. Since these findings were highly suggestive of a malignant tumor, an aspiration biopsy cytology (ABC) was performed. An ABC analysis showed a poor specimen composed only of blood. A sonography-guided core-needle biopsy (CNB) was then performed and was positive for vascular, adipose and mammary tissues, but lacked evidence of a malignancy.

**Figure 3:**
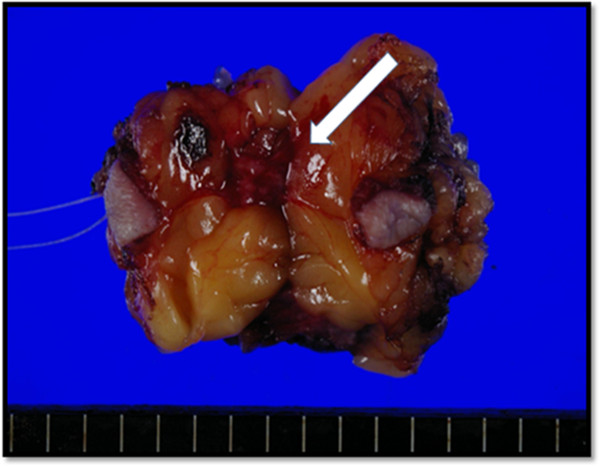
**Macroscopic view of the surgical specimen.**

**Figure 4:**
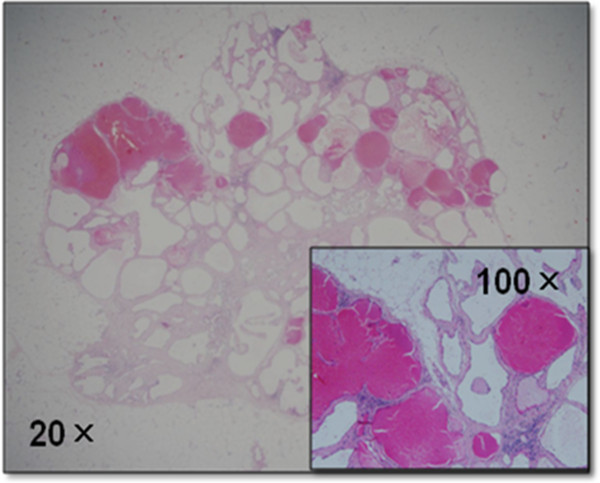
**Hematoxylin and eosin-stained section demonstrated that the tumor consisted of both dilated and congested vessels filled with numerous red blood cells.**

As a result, a total excisional biopsy of the tumor in the inner portion of the right breast was performed under general anesthesia. The resected specimen (17 × 23 mm in size) was a firm, well-circumscribed mass with a brownish hue (Figure 3). A histological examination of the specimen revealed a breast hemangioma lacking cellular atypia (Figure 4). The postoperative course of the disease has remained recurrence-free for 25 months.

## Discussion

Breast hemangioma is a rare, benign vascular tumor representing only 0.4% of all breast tumors [1]. Histologically, breast hemangiomas are classified as either diffuse or localized. Localized hemangiomas are further divided into the following subtypes: peri-lobular, parenchymal, subcutaneous and venous. The parenchymal subtype is classified into capillary and cavernous hemangiomas [2]. Peri-lobular hemangiomas are relatively common lesions that are generally small and impalpable [3]. The other two types of hemangiomas are rare [2]. When making a diagnosis of breast hemangioma, it is important to include hemangiosarcoma, lipoma, cysts, mucinous carcinomas and fibroadenoma in the differential. It should also be noted that an accurate preoperative diagnosis of breast hemangioma seems difficult using current imaging modalities, as well as ABC and CNB.

When looking at summarized patient reports, only three out of 27 breast hemangiomas have been diagnosed preoperatively [4–26]. Furthermore, women comprised 23 of these patients while men accounted for only four (age range 17 to 82 years; mean age 55.6 years). Moreover, the majority of masses in these patients appeared on an MMG as oval, or high-density, lobular lesions with well-circumscribed edges. With sonographic studies, 63.0% of these patients showed well-circumscribed, hypoechoic masses. In addition, vascularity on color Doppler sonography has not been found to have diagnostic value. As previously described, radiographic assistance in reaching the diagnosis has been limited. Breast hemangioma specimens analyzed by CNB or ABC were found to contain hematic material in 40.7% of patients (Additional file 1: Table S1). Unfortunately, these findings are unreliable for making an accurate diagnosis. Once a diagnosis is confirmed, surgical resection is curative since most breast hemangiomas have low malignant potential [3, 27]. Pathologically, it is important to distinguish hemangiomas from hemangiosarcomas. Hemangiosarcomas have an extremely poor prognosis with a three-year survival rate of just 38%. Mesurolle *et al*. reported that CNB was a potential method of distinguishing hemangioma from hemangiosarcoma, even though 37% of hemangiosarcomas are incorrectly diagnosed as hemangiomas using CNB [28, 29]. The difficulty in making a clear distinction between the two is due to the varying degree of cellular atypia found in hemangiosarcomas [30], and supports the need to perform a total excisional biopsy if hemangioma is suspected.

## Conclusions

When ABC or CNB of a suspected breast tumor exhibits only hematic material, it is necessary to consider a vascular tumor in the differential diagnosis. Likewise, it is essential to perform a total excisional biopsy if hemangioma of the breast is suspected.

## Consent

Written informed consent was obtained from the patients for publication of the case report and any accompanying images.

## Electronic supplementary material

Additional file 1: **Reported patients with hemangiomas of breast.** (XLS 35 KB)

## Abbreviations

ABC: aspiration biopsy cytology
CNB: core-needle biopsy
CT: computed tomography
MMG: mammography
MRI: magnetic resonance imaging
US: ultrasonography

## References

[1] Smythe FW (1942). Brief communication: intramammary hemangioma. Ann Surg.

[2] Rosen PP, Jozefczyk MA, Boram LH (1985). Vascular tumors of the breast. IV: the venous hemangioma. Am J Surg Pathol.

[3] Hoda SA, Cranor ML, Rosen PP (1992). Hemangiomas of the breast with atypical histological features: further analysis of histological subtypes confirming their benign character. Am J Surg Pathol.

[4] Sebek BA (1984). Cavernous hemangioma of the female breast. Cleve Clin Q.

[5] Webb LA, Young JR (1996). Case report: haemangioma of the breast–appearances on mammography and ultrasound. Clin Radiol.

[6] Markopoulos C, Sampalis F, Gogas H, Despotidis P, Kyriakou V (1998). Cavernous haemangioma of the breast. A case report. Eur J Gynaecol Oncol.

[7] Vuorela AL (1998). MRI of breast hemangioma. J Comput Assist Tomogr.

[8] Galindo LM, Shienbaum AJ, Dwyer-Joyce L, Garcia FU (2001). Atypical hemangioma of the breast: a diagnostic pitfall in breast fine-needle aspiration. Diagn Cytopathol.

[9] Aurello P, Cicchini C, Mingazzini P (2001). Hemangioma of the breast: an unusual lesion without univocal diagnostic findings. J Exp Clin Cancer Res.

[10] Siewert B, Jacobs T, Baum JK (2002). Sonographic evaluation of subcutaneous hemangioma of the breast. Am J Roentgenol.

[11] Mariscal A, Casas JD, Balliu E, Castellà E (2002). Breast hemangioma mimicking carcinoma. Breast.

[12] Chung SY, Oh KK (2002). Mammographic and sonographic findings of a breast subcutaneous hemangioma. J Ultrasound Med.

[13] Mesurolle B, Wexler M, Halwani F, Aldis A, Veksler A, Kao E (2003). Cavernous hemangioma of the breast: mammographic and sonographic findings and follow-up in a patient receiving hormone-replacement therapy. J Clin Ultrasound.

[14] Hayasaka K, Tanaka Y, Saitoh T, Takahashi M (2003). Gadolinium-enhanced dynamic MRI of breast hemangioma. Comput Med Imaging Graph.

[15] Kinoshita S, Kyoda S, Tsuboi K, Son K, Usuba T, Nakasato Y, Kashiwagi H, Komine K, Takeishi M, Sato S, Takeyama H, Uchida K, Yamazaki Y, Sakamoto G (2005). Huge cavernous hemangioma arising in a male breast. Breast Cancer.

[16] Kim SJ, Han HS, Kim JS, Park JH, Jeon HJ, Yi JG (2006). Cavernous hemangioma of the breast parenchyma with unusual features. J Ultrasound Med.

[17] Vourtsi A, Zervoudis S, Pafiti A, Athanasiadis S (2006). Male breast hemangioma–a rare entity: a case report and review of the literature. Breast J.

[18] Adwani A, Bees N, Arnaout A, Lanaspre E (2006). Hemangioma of the breast: clinical, mammographic, and ultrasound features. Breast J.

[19] Kim SH, Lee JH, Kim DC, Song BJ (2007). Subcutaneous venous hemangioma of the breast. J Ultrasound Med.

[20] Dalfior D, Eccher A, Menestrina F, Bonzanini M, Dvornik G (2008). Epithelioid hemangioma of the thoracic wall mimicking breast tumor: a case report. Breast J.

[21] Leddy R, Cluver A (2010). Mammographic and sonographic characteristics of a cavernous hemangioma in a male patient. J Ultrasound Med.

[22] Kawatra V, Lakshmikantha A, Dhingra KK, Gupta P, Khurana N (2009). A rare coexistence of concurrent breast hemangioma with fibroadenoma: a case report. Cases J.

[23] Ferreira SS, Barra RR, Gonçalves MC, Galvão CN, Santos GC, Alexandrino A, Vieira RA, da Rocha ET, Moriguchi SM (2011). Breast hemangioma investigation–a rare condition documented by nuclear medicine, radiology and pathology. Breast J.

[24] Tilve A, Mallo R, Pérez A, Santiago P (2012). Breast hemangiomas: correlation between imaging and pathologic findings. J Clin Ultrasound.

[25] Ameen R, Mandalia U, Marr AA, McKensie P (2012). Breast Hemangioma: MR Appearance with Histopathological Correlation. J Clin Imaging Sci.

[26] Tadakoshi M, Ishibashi H, Orimoto Y, Sugimoto I, Iwata H, Yamada T, Hida N, Ohta T (2012). Huge hemangioma in the chest mimicking a breast tumor: report of a case. Ann Vasc Dis.

[27] Jozefczyk MA, Rosen PP (1985). Vascular tumors of the breast. II: perilobular hemangiomas and hemangiomas. Am J Surg Pathol.

[28] Mesurolle B, Sygal V, Lalonde L, Lisbona A, Dufresne MP, Gagnon JH, Kao E (2008). Sonographic and mammographic appearances of breast hemangioma. Am J Roentgenol.

[29] Chen KT, Kirkegaard DD, Bocian JJ (1980). Angiosarcoma of the breast. Cancer.

[30] Liberman L, Dershaw DD, Kaufman RJ, Rosen PP (1992). Angiosarcoma of the breast. Radiology.

